# Doctors and Sympathy

**Published:** 1920-09-18

**Authors:** 


### Doctors and Sympathy.

### AN APPRECIATION FROM WALES.

We cannot refrain from quoting from the kindly appreciation in a Welsh newspaper of the personality and benevolence of that notable son of Wales, Dr. Morgan Davies, F.R.C.S., late of Goring Street, London, who died just over a week ago. A good deal of cheap humour is commonly directed against doctors "with a good bedside manner," but there is probably more genuine unobtrusive friendliness in the medical profession than in any other, and the public themselves scarcely realise how important to their well-being, and how practically valuable a thing it is in times of sickness, that the attending doctor should possess the human quality of delicate sympathy, and know how to dispense it.

"Of his writing, his philosophy, his poetry, and his genius," says the writer in the *Western Mail*, referring to Dr. Davies, "the world will soon be told, but I would like to record a few anecdotes I know to be true about him that show at a glance the noble nature of the man himself. For instance, a few years ago he was called to see a very poor young Welshman in Houndsditch. He found him very ill, and very lonely, no friends, and no money wherewith to buy the bare necessaries of life. At once he had him removed to his own house in Goring Street, and under that hospitable roof for six weeks he watched his progress, and under his directions Mrs. Davies nursed him and cared for him night and day until at the end of that time he was fully restored to health, and able to take up his work once more.

"On another occasion he went to see a Welshman of middle age, and discovered by many indirections that his patient was actually ill and suffering from sheer want of food; in fact he was starving! 'Ah,' said the doctor, 'I will send you a box of pills. Yes, bachgen, a box of yellow pills. They will put you on your feet at once!' And so they did, for when the poor fellow received the box he was puzzled as to why it was so heavy, but when the lid was removed he found revealed to his delighted gaze a big pill-box packed to the brim with golden sovereigns.

"It is, of course, well known that he was physician to the Pryces, of Gogerddan, and when he was summoned from London to attend at this historic mansion he naturally took the long railway journey in comfort and as becoming his dignity in a first-class carriage, but as the train got nearer to Wales, and more especially when his ear caught the first homely words in the Welsh language on the stations they passed through, the doctor, with this great love for his countrymen, could tolerate the loneliness of the palatial first-class no longer. Out he would get, and make his way into a third-class compartment among the chattering crowd and the women with their big baskets going to market."

